# Supplementary material for: MetaRibo-Seq measures translation in microbiomes
Source: Nat Commun. 2020 Jun 29;11:3268. doi: 10.1038/s41467-020-17081-z (PMC7324362; doi:10.1038/s41467-020-17081-z)
Supplement: Supplementary file 10 — Supplementary Data 7 [file 41467_2020_17081_MOESM10_ESM.zip › File2/Confidence_High_Taxonomy/12555_out.krona.html]

Javascript must be enabled to view this page.

members
magnitude
magnitudeUnassigned
count
unassigned
taxon
rank

12555\_out

2

superkingdom
2
2

2
phylum
976

200643
class
2

order
171549
2

family
815
1

816
genus
1

SRS1041137\_contig\_number\_12162

1
family
2005525


SRS024625\_contig\_number\_8518
375288
genus
1
